# Stoichiometry and Homeostasis of Sodium and Potassium Underpins Growth Adaptation of *Suaeda salsa* in the Yellow River Delta Wetland

**DOI:** 10.1002/ece3.73687

**Published:** 2026-05-27

**Authors:** Zhang Dongjie, Zhang Kaipeng, Yang Jingyu, Liu Xuepeng, Zhang Lichao, He Wenjun, Zhang Mingye

**Affiliations:** ^1^ Shandong Key Laboratory of Eco‐Environmental Science for the Yellow River Delta Shandong University of Aeronautics Binzhou Shandong Province China; ^2^ Northeast Institute of Geography and Agroecology Chinese Academy of Sciences Changchun Jilin Province China

**Keywords:** allometric growth, coastal wetland, halophyte adaptation, ion homeostasis, sodium‐potassium stoichiometry, soil–plant interface

## Abstract

Sodium (Na) and potassium (K), along with their physiological trade‐offs, play essential roles in maintaining osmotic pressure and ion homeostasis of halophytes inhabiting salt‐alkali wetlands. However, the existence of robust Na‐K homeostasis and the mechanistic linkage of its stoichiometry to plant growth under saline conditions remains poorly understood. Here, we investigated the costal wetlands dominated by halophyte (*Suaeda salsa*) in the Yellow River Delta of China, employing 33 sampling sites to analyze Na‐K stoichiometry and homeostasis across soil and plant tissues, and to assess their effects on plant growth. Results revealed significant differences in Na‐K stoichiometry across soil and plant tissues of *S. salsa* . Leaf Na content and Na:K ratio were 3.08–6.04 times and 2.76–9.37 times higher, respectively, than those in soil and other two tissues, whereas K content of plant tissues was only 49.08%–61.34% of those in soil. Robust and positive homeostasis of Na, K, Na:K ratio, and Na‐K relationships within plant tissues of *S. salsa* . By contrast, soil‐ *S. salsa* interfaces exhibited very few significant relationships for Na, K, Na:K ratio, Na‐K, with K and Na‐K relationships being more robust than the corresponding relationship drawing in Na and Na:K ratio. Notably, while soil Na‐K stoichiometry showed limited correlations with plant density and biomass, tissue Na‐K stoichiometry, particularly in stem Na content and Na:K ratio, was strongly associated with plant height, highlighting their central role in mediating the growth responses of *S. salsa* . These results suggest that *S. salsa* maintains stringent internal Na‐K homeostasis independent of soil variability, with tissue Na‐K stoichiometry serving as a better predictor of plant performance than soil ionicity. The internal Na‐K coordination underpins the ecological adaptation of *S. salsa* in the costal wetlands of the Yellow River Delta.

## Graphical abstract

Na‐K stoichiometry significantly affected by soil and plant tissues of *S. salsa* ; robust plant tissue Na‐K homeostasis contrasts with weak soil–plant ion coupling; coordinated Na‐K regulation underlies salt adaptation in Suaeda salsa.

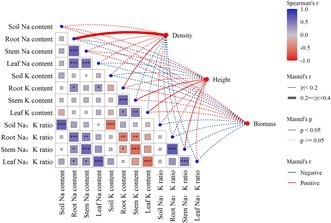

## 1 Introduction

Sodium‐potassium (Na‐K) stoichiometry and homeostasis are integral to the physiological adaptation mechanism of halophytes, significantly influencing plant growth and development in saline wetlands (Adams and Shin 2014; Dai et al. 2025). Coastal saline wetlands, typically characterized by high Na content, constitute a challenging environment for halophyte survival (Jiménez‐Ballesta et al. 2024; Sun et al. 2024). Elevated soil Na content induces osmotic stress and ionic toxicity in plants, impairing key physiological processes and ultimately suppressing growth and reproduction of halophytes (Adams and Shin 2014; Kaleem et al. 2024; Vitt et al. 2020; Sidhoum et al. 2025). Under these conditions, halophytes often modulate internal K content to mitigate the detrimental effects of excess Na, thereby maintaining ionic equilibrium (Barbafieri et al. 2023; Zhao et al. 2022). The regulation of cellular osmotic pressure via Na and K is critical for facilitating the uptake, transport, and allocation of water, nutrient, other resource, these processes being essential for sustaining plant growth under high salinity (Cheng et al. 2020; Kaleem et al. 2022). Previous studies have elucidated various aspects of Na‐K physiology, including ion absorption and transport mechanisms, Na^+^:K^+^ balance, osmotic regulation, salt tolerance strategies, oxidative stress mitigation, and molecular controls (Adams and Shin 2014; Cheng et al. 2020; Guo et al. 2023; Wegner et al. 2025; Yasseen and Al‐Thani 2022). The maintenance of Na‐K homeostasis in plants relies on a suite of integrated physiological and molecular mechanisms, including the regulated expression and activity of key transporter proteins, the central signaling role of Ca^2+^ and associated CBL‐CIPK networks, coordinated hormonal and reactive oxygen species signaling, the involvement of compatible solutes in energy and osmotic adjustment, and dynamic cellular compartmentalization and ion redistribution processes (Feng et al. 2025; Leigh 2001; Anil Kumar et al. 2024). Collectively, these studies underscore that Na‐K stoichiometry and balance exert broad influences on plant growth that extend beyond mere regulatory functions (Adams and Shin 2014; Srivastava et al. 2020; Walker et al. 1996). Nevertheless, it remains unclear whether systematic Na‐K homeostasis, especially the homeostasis of total Na and K contents, exists across the plant–soil continuum, and how such relationships can be quantitatively assessed (e.g., using Linear or Power models) in dynamic coastal wetland ecosystems.

Plant Na‐K homeostasis refers to the capacity of plant systems from cellular to whole‐organism levels to maintain a physiologically appropriate and dynamic Na‐K balance under environmental stressors such as salinity, drought, and oxidative stress, mediated through integrated physiological, molecular, and signaling networks (Feng et al. 2025; Walker et al. 1996; Wegner et al. 2025). Both Na and K are essential for the growth and life‐cycle completion of halophyte, and sustaining a stable cytoplasmic Na:K ratio is critical for normal metabolic function, optimal development, and ultimately, ecosystem stability (Johnson et al. 2022; Zhao et al. 2022; Zuo et al. 2025). Previous research has highlighted that plant elemental stoichiometry is jointly shaped by soil nutrient availability and plant nutritional demand, a conceptual framework that effectively bridges plant physiology and soil ecology (Pan et al. 2020; Zuo et al. 2024, 2025). This systemic perspective accounts for bidirectional plant–soil interactions and offers a unified basis for interpreting coordinated shifts among multiple elements (Srivastava et al. 2020; Zuo et al. 2025). The framework not only explains adaptive plant responses to edaphic conditions and cross‐ecosystem variation in elemental ratios based on the total content of some certain elements, but also aligns with established theories such as the Growth Rate Hypothesis (Adams and Shin 2014; Pan et al. 2020). Moreover, it exhibits considerable predictive power by enabling estimations of plant elemental composition from soil element data (Zuo et al. 2025). Supported by extensive empirical evidence across spatial scales and plant functional types, the approach holds practical relevance for applications in ecological restoration (Pan et al. 2020; Zhang, Xia, et al. 2023). Importantly, it underscores the active regulatory role of plants in elemental composition‐ a perspective that carries greater explanatory value than purely environment‐driven determinism (Guo et al. 2023; Johnson et al. 2022; Zuo et al. 2025). Nevertheless, the framework exhibits several limitations. It tends to oversimplify by neglecting key rhizosphere processes, internal plant nutrient translocation and partitioning mechanisms, and non‐chemical drivers such as climatic variables and biotic interactions (Anil Kumar et al. 2024; Feng et al. 2025; Pan et al. 2020). Mechanistically, it lacks detail regarding how plants perceive and regulate ion uptake, how elements interact synergistically, and how genetic controls modulate these processes (Guo et al. 2023; Almeida et al. 2017). Furthermore, it insufficiently accounts for specialized adaptive strategies in particular plant groups or non‐equilibrium system behaviors following disturbances or under rapid environmental change (Johnson et al. 2022; Zhao et al. 2022). Challenges persist in operationally defining and quantifying soil nutrient availability and plant demand disentangling correlation from causation, and distinguishing between soil‐imposed limitations and plant‐mediated regulatory outcomes (Wegner et al. 2025; Zuo et al. 2024). Therefore, this study focuses on the intra‐plant allocation mechanism of Na and K, their synergistic role in maintaining Na‐K homeostasis, and the homeostatic strategies of halophytes in dynamic stress environments, as revealed by total Na and K contents (as opposed to specific ionic state). These investigations hold significant theoretical value.

The Yellow River Delta wetland represents one of the best preserved warm temperate wetland ecosystems in China and serves as a global archetype of newly formed estuary wetlands (Bai et al. 2020; Han et al. 2018; Zhang et al. 2026). This region encompasses a complex of wetland ecosystems distributed along the Yellow River's ecological corridor, constituting a critical ecological zone within the delta and intersecting with nationally significant coastal wetland systems (Shao et al. 2022; Liu, Fagherazzi, et al. 2024). As a pivotal component of the Yellow River Basin's wetland spatial network, it is recognized as a priority area for both regional wetland conservation and national ecological security (Yu et al. 2024). In recent decades, increasing soil salinization driven by climate change and anthropogenic activities has posed considerable threats to the ecological security of the region (Li et al. 2023; Ren et al. 2024). These changes have heightened interest in the adaptive responses of halophytes to saline stress, particularly in the context of wetland protection and restoration (Zhao et al. 2022; Sun et al. 2024; Wang, Wu, et al. 2025). Many studies have documented physiological and ecological adaptations of halophytes under wetland degradation and restoration, often under controlled treatments such as varying hydrology, salinity, or nutrient regimes (Luo et al. 2025; Wang, Wu, et al. 2025; Zhao et al. 2022), and some have focused on the role of Na‐K stoichiometry in mediating salt tolerance and osmotic regulation of halophytes in coastal saline wetlands (Adams and Shin 2014; Wegner et al. 2025; Sardans and Peñuelas 2021). Critically, the Na‐K stoichiometry and homeostasis across the soil–plant interface and its functional implications for halophyte growth remain poorly understood, representing a key knowledge gap in understanding plant adaptation mechanisms in these vulnerable ecosystems of Yellow River Delta.

*Suaeda salsa* is a representative halophyte in the coastal wetlands of the Yellow River Delta, characterized by high salt tolerance, rapid growth, strong reproductive capacity, and remarkable adaptability to saline conditions (Zhao et al. 2022; Zhang, Li, et al. 2023; Sun et al. 2024). Furthermore, *S. salsa* plays a critical ecological role in reducing soil salinity through salt accumulation, thereby improving edaphic conditions for the establishment and growth of other wetland plant species (Guan et al. 2011; Wang and Liu 2024; Zhao et al. 2024). Owing to these traits, it is considered a key species for ecological restoration and salinity management in coastal wetlands. Previous studies have documented how variable salinity, hydrological regimes, and nitrogen deposition affect the physiological and ecological performance of *S. salsa* in costal wetlands (Guan et al. 2011; Zhang et al. 2020), as well as the efficacy of restoration strategies such as hydrological connectivity enhancement, micro‐topography modification, and freshwater irrigation for salt suppression (Wang et al. 2022; Zhang, Xia, et al. 2023; Zhang, Zhang, et al. 2023). Many of these responses can be attributed to the regulation of Na and K, encompassing ion uptake and translocation, osmotic adjustment, oxidative stress mitigation, and molecular control of ion homeostasis (Guan et al. 2011; Zhang et al. 2020; Zhang, Xia, et al. 2023; Zhang, Zhang, et al. 2023). Nevertheless, few studies have quantitatively examined Na‐K stoichiometry and homeostasis in relation to growth performance of *S. salsa* , nor have they applied systemic modeling approaches (e.g., mechanistic or allometric models) to investigate Na‐K homeostasis within the soil‐ *S. salsa* continuum in the Yellow River Delta wetlands.

To address these knowledge gaps, we investigated *S. salsa* , a dominant halophytic species in the coastal wetlands of Yellow River Delta, to examine the stoichiometry and homeostasis of total Na and total K contents within the plant–soil system (across plant tissues and at the plant–soil interface), and to evaluate its influence on plant growth. Specifically, we aim to address the following questions: (1) How does Na‐K stoichiometry vary between soil and different plant tissues of *S. salsa* ? (2) Does Na‐K homeostasis exist within the *S. salsa* ‐soil system, and is it more robust within plant tissues or at the plant–soil interface? (3) Is Na‐K stoichiometry significantly correlated with the growth of *S. salsa* ? We hypothesized that: (1) Na content and Na:K ratio would increase progressively from soil to root, stem, and leaf tissues, whereas K content would decrease, reflecting ion allocation strategies and salt tolerance mechanisms of *S. salsa* ; (2) due to ion concentration gradients and selective transport under saline conditions, the Na‐K stoichiometry of *S. salsa* would be influenced by soil ion availability, yet Na‐K homeostasis within plant tissues would be more strongly regulated than at the soil–plant interface; and (3) the growth of *S. salsa* would be closely associated with both soil and tissue Na‐K stoichiometry.

## 2 Materials and Methods

### 2.1 Study Area

This study was conducted in the costal wetlands of Yellow River Delta, Shandong Province, China (37°20′ N–38°12′ N, 118°01′ E–119°13′ E). The region forms a fan‐shaped region with its apex at Ninghai in Kenli County, extending north to the Tiao'er River estuary and south to the Xiaoqing River estuary (Zhang et al. 2026). The climate is characterized as a warm temperate continental monsoon, with distinct seasonal variations and a mean annual temperature of 12.9°C. Annual precipitation ranges from 550 to 640 mm, while annual evaporation ranges between 1900 and 2200 mm. The soils are predominantly fluvo‐aquic, salinized fluvo‐aquic, and coastal saline alluvial, largely shaped by sedimentation processes of the Yellow River. This region is ecologically fragile and supports dominant wetland plant communities including *Phragmites australis* , *Tamarix chinensis* , and *Suaeda salsa*. *S. salsa* is a key pioneer species in these coastal wetlands and plays an essential role in the ecological restoration of the Yellow River Delta (Guan et al. 2011; Yu et al. 2024; Sun et al. 2020).

### 2.2 Samples Collection and Determination

A total of 33 sites dominated by *S. salsa* were established across coastal wetlands near the Tiao'er River estuary (11 sites) and within the Yellow River Delta National Nature Reserve (22 sites) during July to August 2024 (the peak season of plant growth). At each site, the height and density of *S. salsa* (# m^−2^) were recorded. The plant height is measured by a tape measure, and the plant density is obtained by manually counting within the sample plot. Aboveground and belowground biomass of *S. salsa* and underlying soil (0–20 cm depth) were collected from a 50 cm × 50 cm quadrat, placed in sealed bags with ice packs, labeled, and transported to the Shandong Key Laboratory of Eco‐Environmental Science for the Yellow River Delta in Shandong University of Aeronautics for processing. Plant samples of *S. salsa* were thoroughly rinsed to remove soil and debris, separated into root, stem, and leaf, and placed in pre‐labeled paper bags. Tissues were oven‐killed at 120°C for 3 h and then dried at 75°C to constant weight for biomass determination. Soil samples were air‐dried, and all samples were finely ground to pass through a 1 mm sieve for subsequent analysis.

To determine total Na content, powdered samples were digested with aqua regia and perchloric acid to mineralize organic matter and dissolve silicate structures, followed by filtration and dilution to a constant volume to obtain a quantifiable solution (Bao 2000). For total K content analysis, samples were fused with NaOH, dissolved in acid, filtered, and diluted to a fixed volume to prepare a test solution (Bao 2000). The total Na and K contents in the digested solutions were quantified using flame photometry. The Na:K ratio was calculated based on the measured total Na and K contents in each soil and plant tissue sample (root, stem, and leaf). Soil total C content and N contents were determined using a Vario EL III elemental analyzer. Total P content was quantitatively analyzed by molybdenum‐antimony spectrophotometric method after melting with NaOH (Bao 2000). Soil stoichiometric ratios (C:N, C:P, and N:P) were calculated based on the corresponding C, N, and P contents, and expressed as mass ratios. Soil key physicochemical properties of the studied coastal wetlands were shown in Table 1 based on all data.

**TABLE 1:** Soil physicochemical properties at the sampling sites (mean ± standard error).

Soil properties	
Soil bulk density (g/cm^3^)	1.33 ± 0.13
Soil salt content (%)	12.23 ± 6.66
Soil water content (%)	34.96 ± 7.46
Total C content (%)	1.68 ± 0.46
Total N content (%)	0.07 ± 0.05
Total P content (mg/g)	0.61 ± 0.15
Total Na content (mg/g)	17.27 ± 0.24
Total K content (mg/g)	17.46 ± 0.27
C:N ratio	40.21 ± 59.11
C:P ratio	27.79 ± 6.54
N:P ratio	1.15 ± 0.67
Na:K ratio	1.00 ± 0.02

### 2.3 Statistical Analyses

Statistical analyses were conducted to examine compartment‐specific differences in total Na and K contents and their stoichiometric ratios across the soil‐ *S. salsa* system (soil, root, stem, and leaf). Before further analyses, normality (Shapiro–Wilk test) and homogeneity of variance (Levene's test) were assessed for total Na and K contents, along with their ratios. When data met the assumptions of normality and homoscedasticity, a one‐way ANOVA followed by Duncan's post hoc test (*α* = 0.05) was applied to assess significant differences among different compartments. For variables violating the homogeneity assumption (e.g., K content and Na:K ratio), a permutational ANOVA was performed using the lmPerm package in R 4.3.1, which provides robust inference under heteroscedastic conditions without relying on parametric variance assumptions. Homeostasis of Na, K, and Na:K ratio was evaluated between plant tissues (root‐stem, root‐leaf, stem‐leaf) and across soil–plant compartments (whole plant–soil, root‐soil, stem‐soil, leaf‐soil) using power models ($y={\mathrm{ax}}^{b}$) (Pan et al. 2020; Zuo et al. 2025). Additionally, the stoichiometric homeostasis between Na and K contents was examined within both soil and plant tissues. Finally, regression analyses were performed to quantify the responses of plant density, height, and biomass to variations in soil and tissue Na‐K stoichiometry.

## 3 Results

### 3.1 Na‐K Stoichiometry Across Soil‐ *S. salsa* Compartments

**FIGURE 1:**
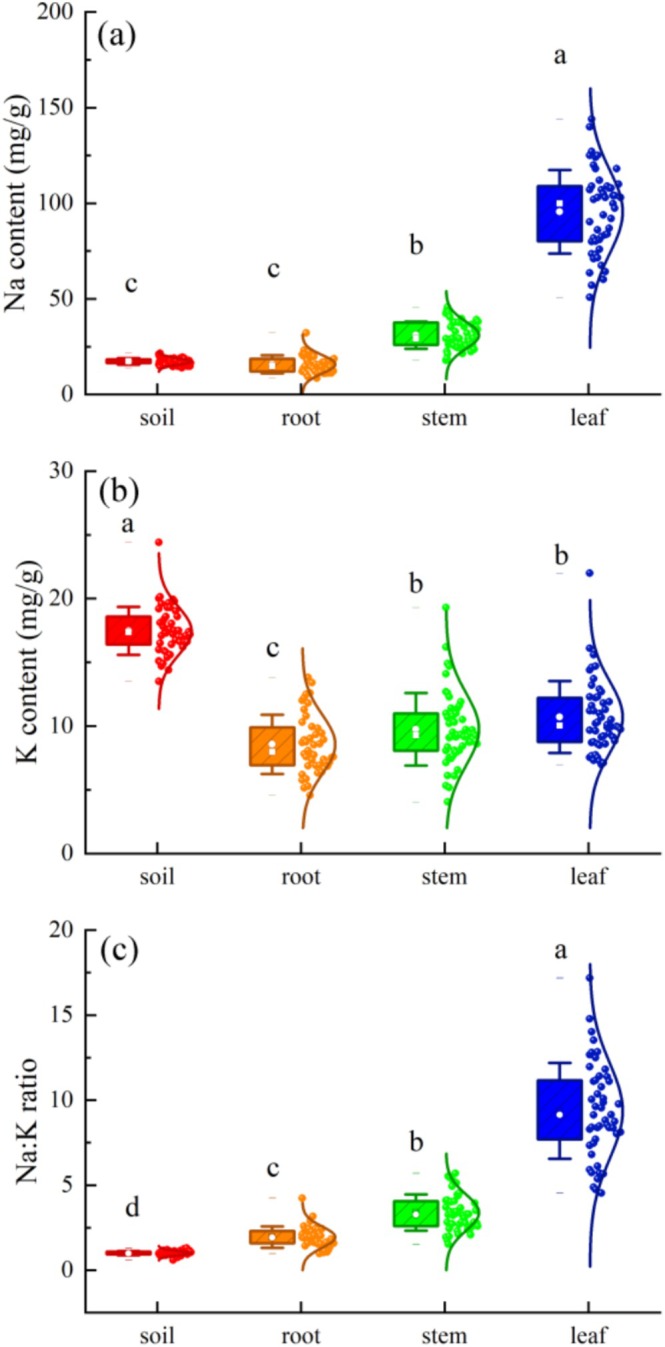
The Na‐K stoichiometry ((a) Na; (b) K; (c) Na:K ratio) in soil and plant tissues of *Suaeda salsa* in the Yellow River Delta wetlands. Different lowercase letters indicate significant differences (*p* < 0.05) in Na‐K stoichiometry of *S. salsa* among soil and plant tissues.

Total Na and K concentrations and their ratios exhibited significant differences across soil and *S. salsa* compartments in the Yellow River Delta wetlands (Figure 1, *p* < 0.001). Na content increased progressively from soil to root, stem, and leaf tissues, with leaf Na content (95.57 mg/g) being significantly greater than that in stems, roots, and soil (*p* < 0.001). In contrast, K content of plant tissues represented only 49.08%–61.34% of soil K content, and the Na:K ratio in plant tissues increased 1.94–9.37 times of soil.

### 3.2 Na‐K Homeostasis in Soil‐ *S. salsa* Continuum

**FIGURE 2:**
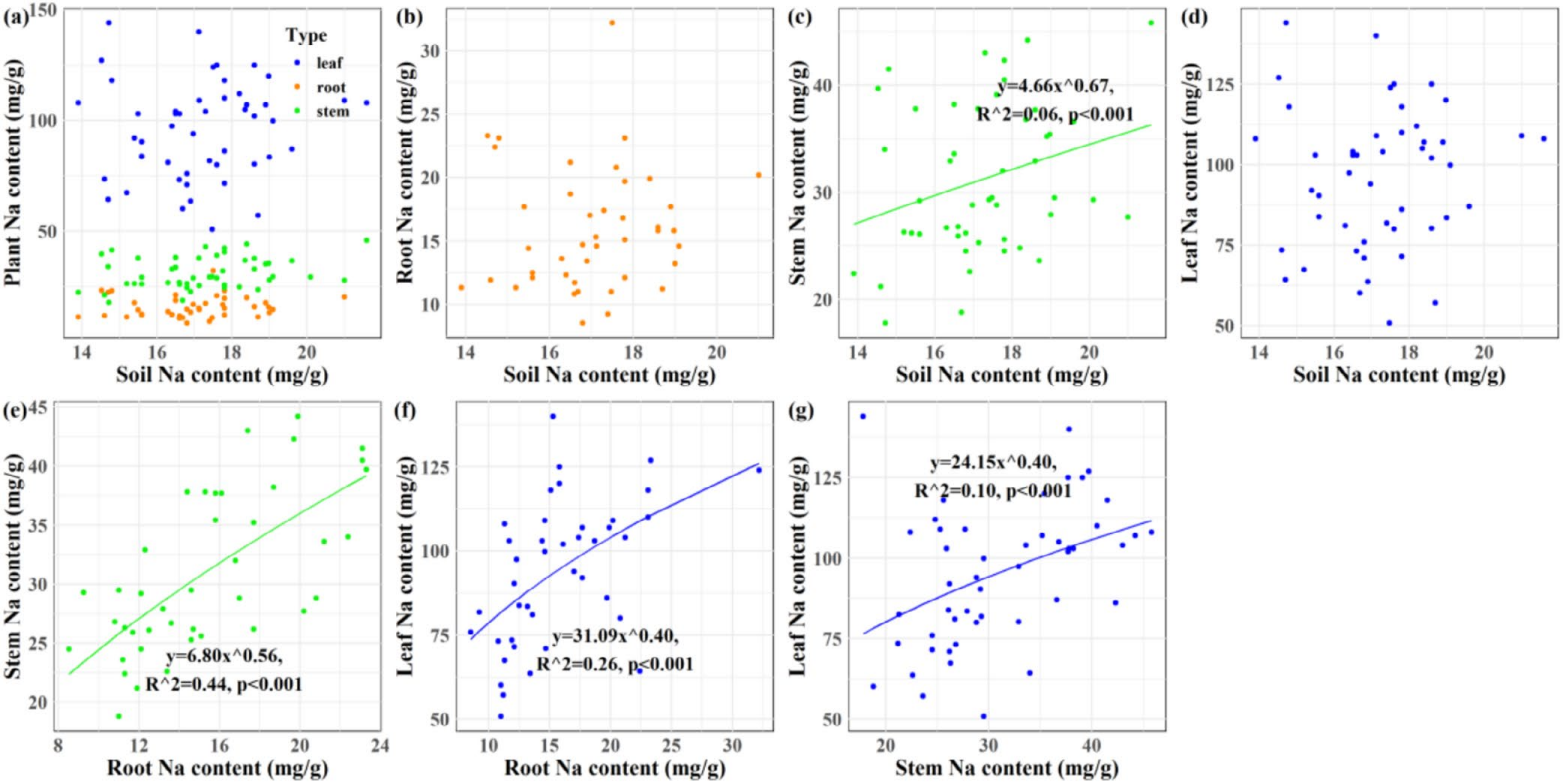
The Na homeostasis in soil‐ *S. salsa* continuum in the Yellow River Delta wetlands. (a) Plant‐soil Na homeostasis; (b) Root‐soil Na homeostasis; (c) Stem‐soil Na homeostasis; (d) Leaf‐soil Na homeostasis; (e) Stem‐root Na homeostasis; (f) Leaf‐root Na homeostasis; (g) Leaf‐stem Na homeostasis.

**FIGURE 3:**
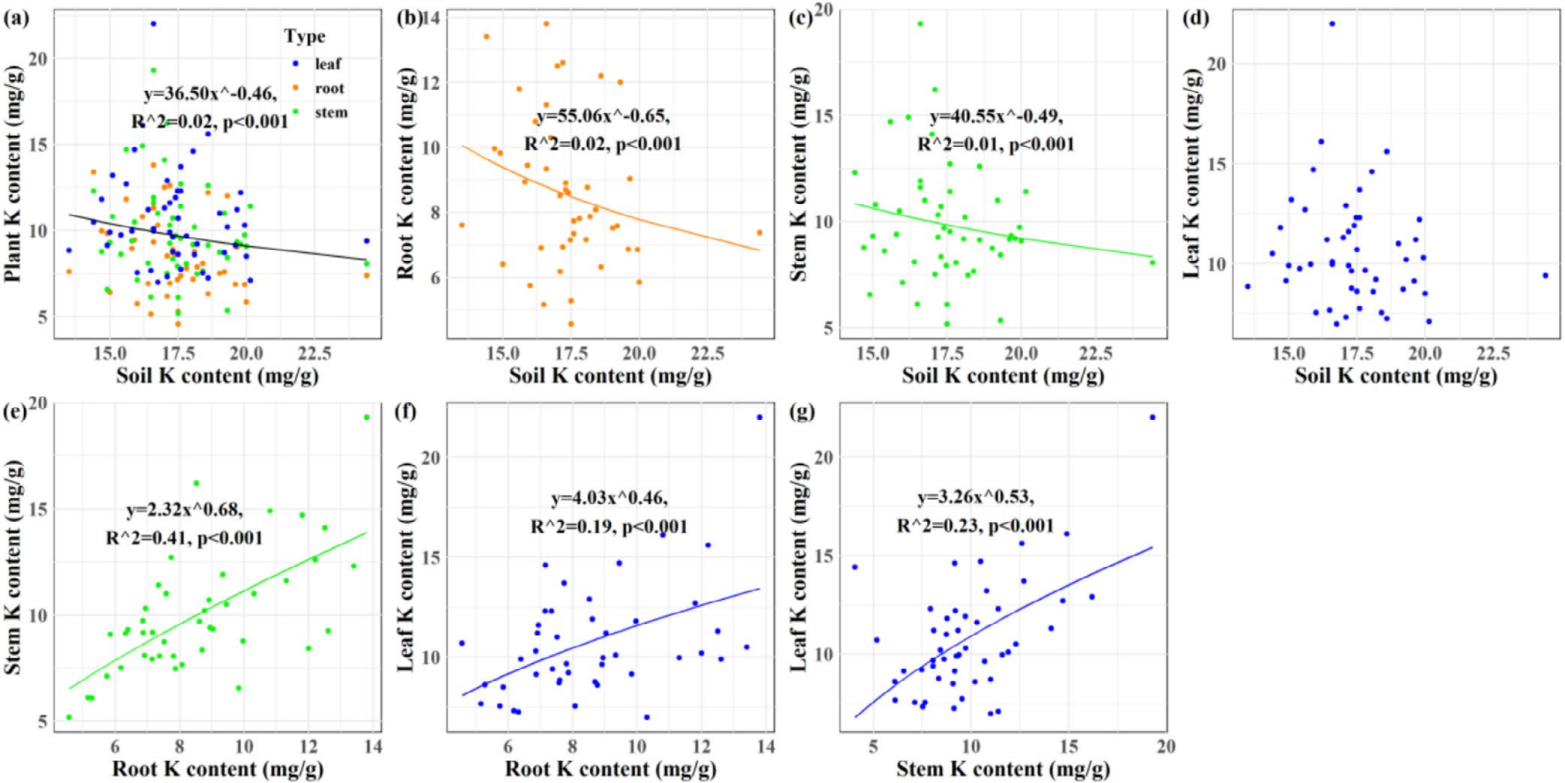
The K homeostasis in soil‐ *S. salsa* continuum in the Yellow River Delta wetlands. (a) Plant‐soil K homeostasis; (b) root‐soil K homeostasis; (c) stem‐soil K homeostasis; (d) leaf‐soil K homeostasis; (e) stem‐root K homeostasis; (f) leaf‐root K homeostasis; (g) leaf‐stem K homeostasis.

**FIGURE 4:**
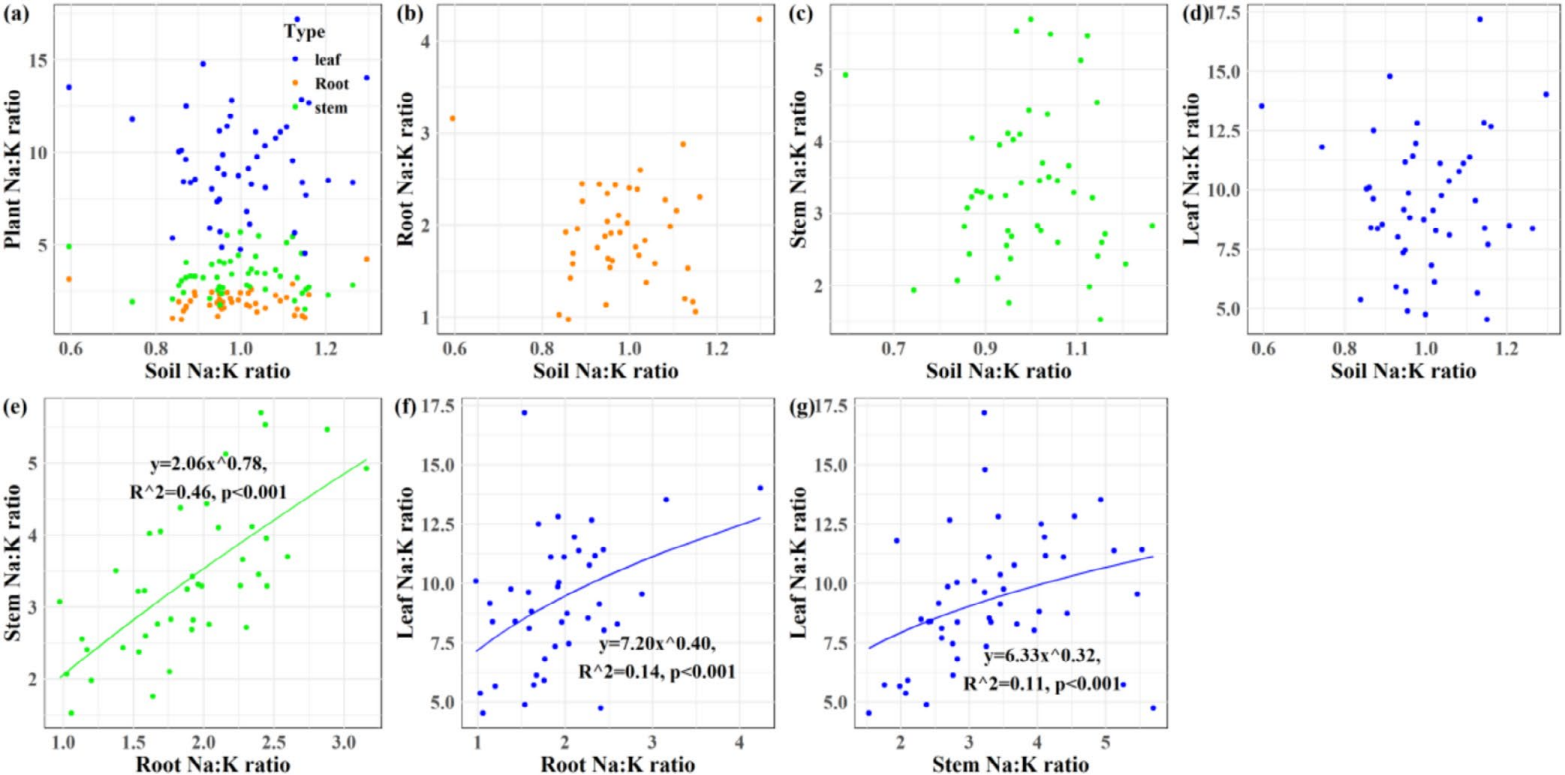
Na:K homeostasis in soil‐ *S. salsa* continuum in the Yellow River Delta wetlands. (a) Plant‐soil Na:K ratio homeostasis; (b) root‐soil Na:K ratio homeostasis; (c) stem‐soil Na:K ratio homeostasis; (d) leaf‐soil Na:K ratio homeostasis; (e) stem‐root Na:K ratio homeostasis; (f) leaf‐root Na:K ratio homeostasis; (g) leaf‐stem Na:K ratio homeostasis.

**FIGURE 5:**
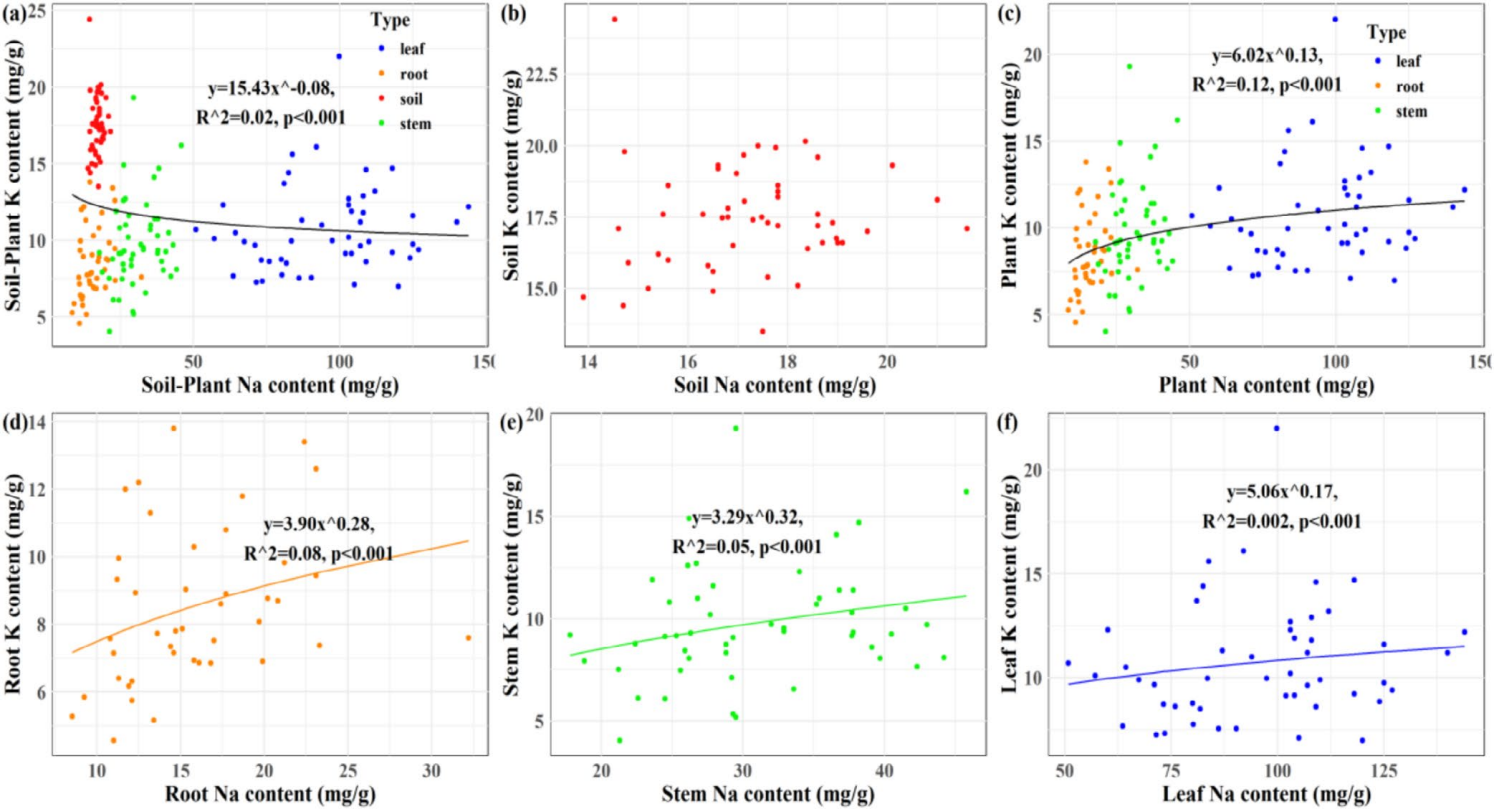
The Na‐K homeostasis within *Suaeda salsa* tissues and at soil–plant interface in the Yellow River Delta wetlands. (a) Plant‐soil Na‐K homeostasis; (b) soil Na‐K homeostasis; (c) plant Na‐K homeostasis; (d) root Na‐K homeostasis; (e) stem Na‐K homeostasis; (f) leaf Na‐K homeostasis.

A significant Na homeostatic relationship was observed specifically between stem and soil (*p* < 0.001; Figure 2). Robust positive Na homeostasis was identified among plant tissues (*p* < 0.001; Figure 2e–g). For potassium, significant negative homeostasis was observed at the soil–plant interface, except in leaf‐soil pairs (*p* < 0.001; Figure 3). Inter‐tissue K homeostasis remained positive (p < 0.001; Figure 3e–g). No significant Na:K homeostasis was found between soil and plant compartments (Figure 4a–d), whereas positive stoichiometric homeostasis occurred among plant tissues (*p* < 0.001; Figure 4e–g). Significant negative Na‐K homeostatic relationships were identified in the soil–plant continuum (*p* < 0.001; Figure 5a). By contrast, positive Na‐K homeostasis was evident within plant tissues (*p* < 0.001; Figure 5c–e).

### 3.3 Responses of Plant Density, Height and Biomass of *Suaeda salsa* to Soil and Plant Na:K Stoichiometry

**FIGURE 6:**
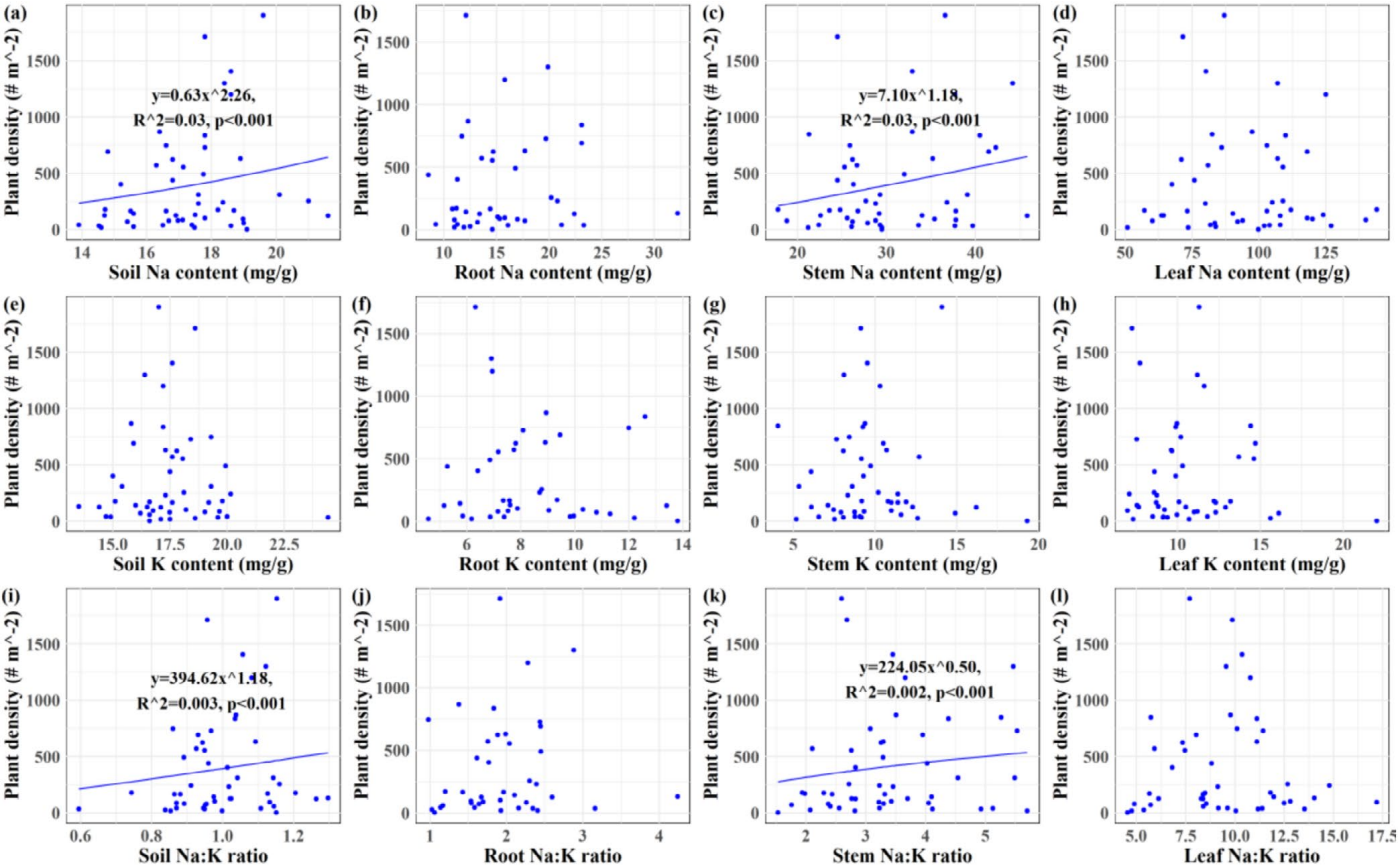
Relationships between *Suaeda salsa* density and Na‐K stoichiometry in plant–soil continuum in the Yellow River Delta wetlands. (a) Plant density‐soil Na content; (b) plant density‐root Na content; (c) plant density‐stem Na content; (d) plant density‐leaf Na content; (e) plant density‐soil K content; (f) plant density‐root K content; (g) plant density‐stem K content; (h) plant density‐leaf K content; (i) plant density‐soil Na:K ratio; (j) plant density‐root Na:K ratio; (k) plant density‐stem Na:K ratio; (l) plant density‐leaf Na:K ratio.

**FIGURE 7:**
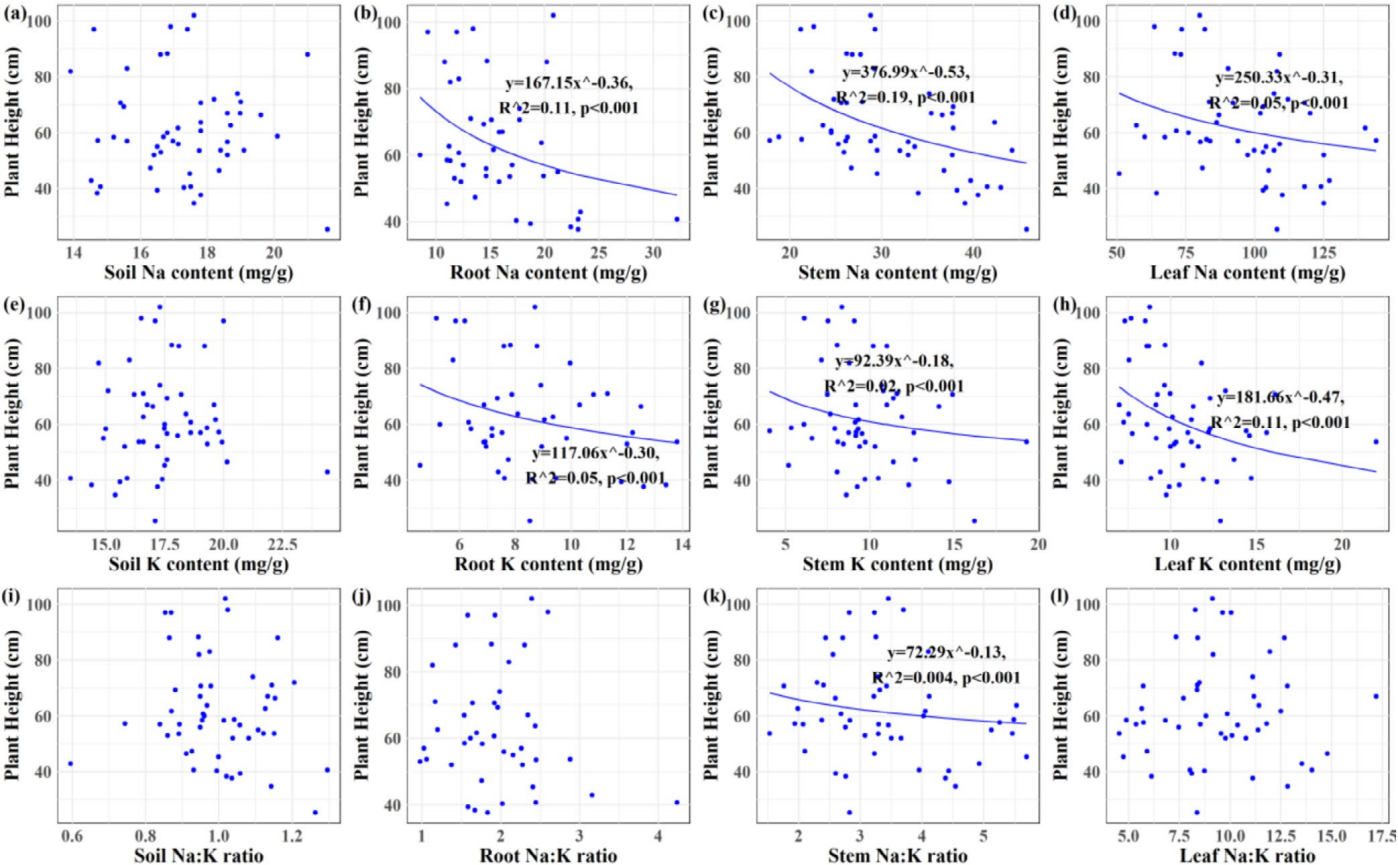
The relationships between plant height of *Suaeda salsa* and Na‐K stoichiometry in plant–soil continuum of the Yellow River Delta wetlands. (a) Plant height‐soil Na content; (b) plant height‐root Na content; (c) plant height‐stem Na content; (d) plant height‐leaf Na content; (e) plant height‐soil K content; (f) plant height‐root K content; (g) plant height‐stem K content; (h) plant height‐leaf K content; (i) plant height‐soil Na:K ratio; (j) plant height‐root Na:K ratio; (k) plant height‐stem Na:K ratio; (l) plant height‐leaf Na:K ratio.

**FIGURE 8:**
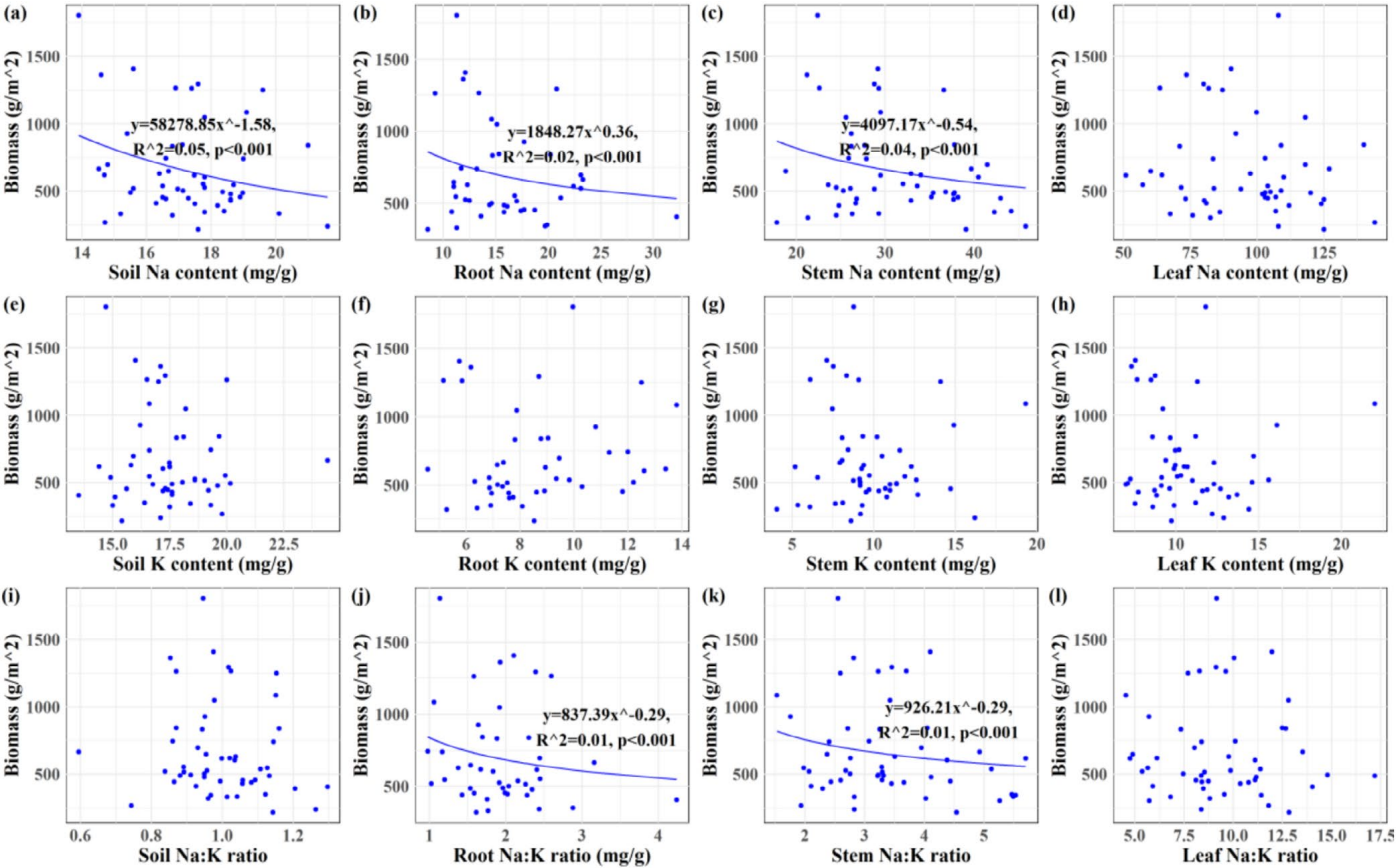
The relationships between biomass of *Suaeda salsa* and Na‐K stoichiometry in plant–soil continuum of the Yellow River Delta wetlands. (a) Biomass‐soil Na content; (b) biomass‐root Na content; (c) biomass‐stem Na content; (d) biomass‐leaf Na content; (e) biomass‐soil K content; (f) biomass‐root K content; (g) biomass‐stem K content; (h) biomass‐leaf K content; (i) biomass‐soil Na:K ratio; (j) biomass‐root Na:K ratio; (k) biomass‐stem Na:K ratio; (l) biomass‐leaf Na:K ratio.

Plant density of *S. salsa* exhibited a positive correlation with soil Na content, stem Na content, soil Na:K ratio, and stem Na:K ratio (*p* < 0.001; Figure 6a,c,i,k). Plant height was negatively correlated with Na and K contents in root, stem, and leaf tissues (*p* < 0.001), as well as with stem Na:K ratio (*p* < 0.001; Figure 7b–d,f–h,k). Plant biomass of *S. salsa* declined with increasing Na contents in soil, roots, and stems, and with elevated Na:K ratios in root and stem (*p* < 0.001; Figure 8a–c,j–k).

### 3.4 Correlation Analysis

**FIGURE 9:**
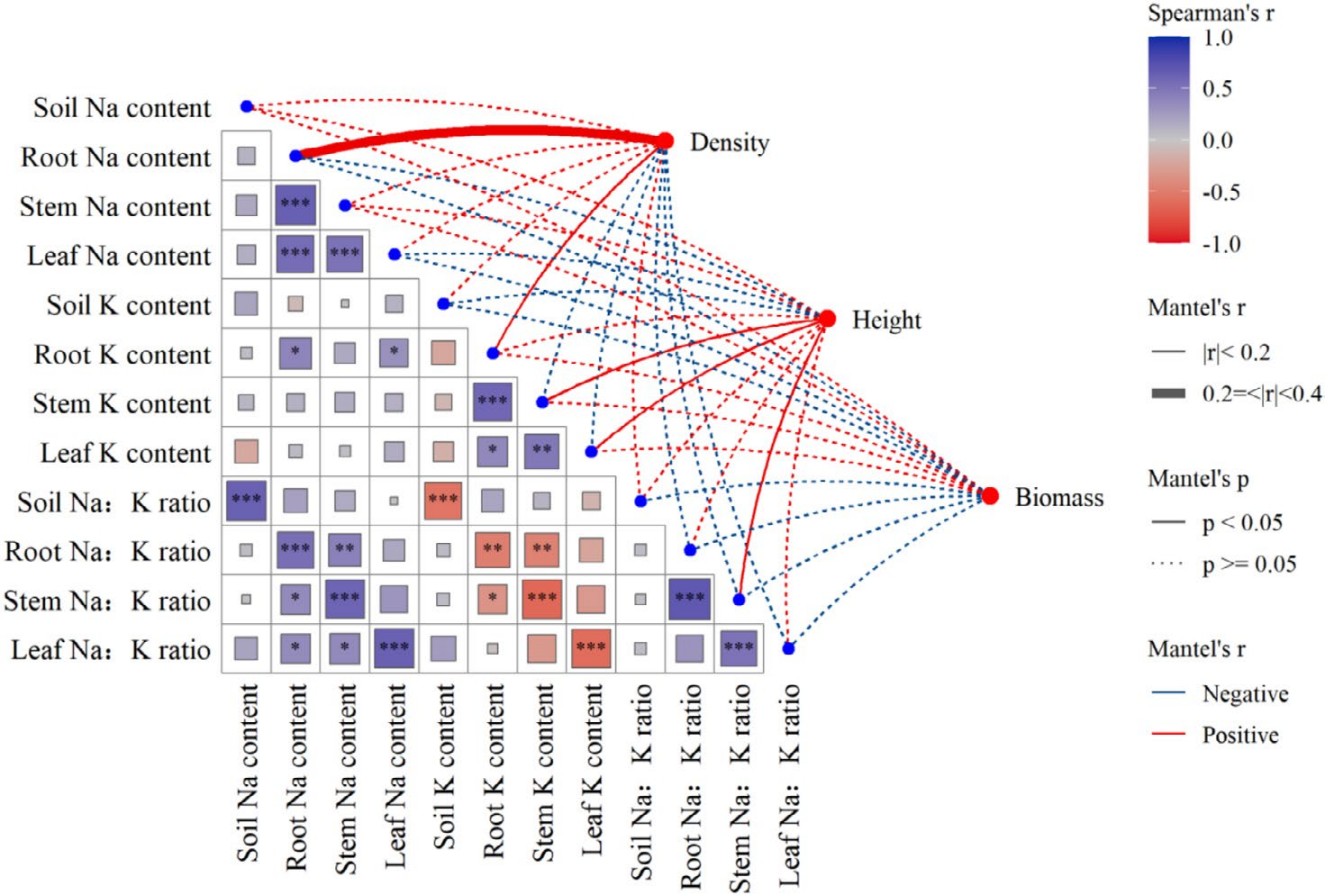
Spearman correlation analysis and Mantel test of height, density, biomass, Na‐K stoichiometry of *Suaeda salsa* in the Yellow River Delta wetlands. *p < 0.05; **p < 0.01; ***p < 0.001.

Significant positive correlations were observed between tissue Na contents, tissue K contents, as well as between tissue Na:K ratios (except root‐leaf Na:K ratio, Figure 9). Root Na and leaf Na contents were significantly correlated with root K content. Density of *S. salsa* was significantly linearly correlated with root Na content and root K content. Plant height exhibited significant linear relationships with stem and leaf K contents, and stem Na:K ratio. However, soil Na‐K stoichiometry is a poor predictor for plant growth.

## 4 Discussion

### 4.1 Ion Compartmentalization and Coordinated Na‐K Regulation Underlie Salt Tolerance and Adaption in *Suaeda salsa*

*Suaeda salsa* is a typical salt‐accumulating halophyte that can actively absorb and accumulate salts from the soil, mitigating internal salt content through water storage in its fleshy leaves (Zhao et al. 2022; Guan et al. 2011). Consistent with our first hypothesis, Na content increased progressively from root to stem and leaf (Figures 1a and 9), forming a distinct ion concentration gradient across tissues. The absence of significant differences in Na content between soil and root suggests the establishment of an Na equilibrium at the soil‐root interface, supporting stable water uptake potential (Ding et al. 2024; Li, Zhang, et al. 2024; Zhang et al. 2020). Elevated Na content in root enhances osmotic potential, facilitating water acquisition from saline soils (Adams and Shin 2014; Vaziriyeganeh et al. 2022). This internal Na gradient in *Suaeda salsa*, coupled with transpiration‐driven water flow, enables efficient water transport to leaves for salt dilution and redistribution, thereby mitigating Na^+^ toxicity (Naz et al. 2023; Vitt et al. 2020; Zhang, Xia, et al. 2023; Zhang, Zhang, et al. 2023). Additionally, Na accumulation works synergistically with compatible solutes (e.g., proline and betaine) to maintain cellular osmotic balance under saline conditions (Zhang, Xia, et al. 2023; Zhang, Zhang, et al. 2023; Sapiña‐Solano et al. 2024).

The uneven distribution of Na across plant tissues of *S. salsa* indicates active compartmentalization, a key adaption strategy for managing ion homeostasis (Adams and Shin 2014; Lugo et al. 2024; Naz et al. 2023). *S. salsa* sequesters excess Na into vacuoles via vacuolar membrane Na^+^/H^+^ antiporters, minimizing Na toxicity in the cytoplasm, while exploiting Na^+^ for osmotic adjustment (Guo et al. 2023; Li et al. 2025). Under high Na conditions, this mechanism limits Na^+^ influx and enhances cytoplasmic detoxification (Lugo et al. 2024; Li, Xu, et al. 2024; Li et al. 2025). Such compartmentalization is critical for maintaining essential physiological processes, including electrochemical gradients, cell signaling, osmotic regulation, and energy metabolism (Adams and Shin 2014; Li et al. 2025).

K is an essential nutrient that plays a critical role in plant growth by regulating osmotic balance, enzyme activation, metabolic processes, photosynthesis, stress tolerance, and yield formation (Cheng et al. 2020; Srivastava et al. 2020; Walker et al. 1996). As a major osmotic regulator, K controls stomatal aperture, reduces water loss, and enhances drought resistance by maintaining cellular K homeostasis (Adams and Shin 2014; Wegner et al. 2025). In *S. salsa* , K works synergistically with Na to improve root water uptake and mitigate water stress under saline conditions. In this study, soil K content is significantly higher than that in plant tissues (*p* < 0.001, Figures 2b and 9). Under saline conditions, root cells are depolarized, making passive (AKT channel‐mediated) K^+^ uptake not possible (Chakraborty et al. 2016; Shabala et al. 2016), forcing plants to rely on high‐affinity uptake systems that are much less effective. Membrane depolarization also triggers massive K^+^ loss via depolarization‐activated outward rectifying GORK channels (Wu et al. 2018; Shabala 2017). *S. salsa* exhibits increasing K content from root to leaf alongside a steep rise in Na:K ratio (Figures 2b–c and 9), and the main reason for this is depolarization‐triggered K^+^ loss via outward rectifying GORK channels, as demonstrated in direct experiments using GORK and AKT mutants (Shabala and Cuin 2008).

Beyond its osmotic functions, K stabilizes membrane potential, activates enzymes, and supports structural integrity under stress, underscoring its cytoprotective roles (Li et al. 2019, 2020). Under low K conditions, high‐affinity transporters and permeases help maintain K^+^ homeostasis (Duan et al. 2015; Song et al. 2022). Notably, *S. salsa* may partially substitute K^+^ with Na^+^ for osmotic functions under K^+^ deficiency (Almeida et al. 2017; Walker et al. 1996), a plasticity facilitated by key genes including NHX, SOS1, and HKT1, which coordinately regulate Na‐K distribution and balance (Duan et al. 2015; Wang et al. 2020). Furthermore, salt stress signaling pathways, such as Ca^2+^ transients and ABA, modulate the expression of these ion transporters, ultimately shaping Na‐K stoichiometry and plant adaptation (Mishra et al. 2024; Zhang, Tian, and Mai 2024).

### 4.2 Robust Plant Tissue Na‐K Homeostasis of *Suaeda salsa* Contrasts With Weak Soil–Plant Ion Coupling

Our second hypothesis was partially supported: plant Na content and Na:K ratio were largely uncorrelated with corresponding soil Na‐K traits, with the exception of a significant stem‐soil Na relationship (Figures 2, 3, 4, 5, 6 and 9). Conversely, elevated soil K content was associated with reduced K accumulation in *S. salsa* , though not in leaf. This finding contrasts with controlled studies reporting a positive correlation between external NaCl concentration and tissue Na accumulation (Vitt et al. 2020). The poor predictive power of soil Na‐K stoichiometry for plant tissue composition can be attributed to the suite of specialized cation uptake systems in roots, which function under distinct regulatory mechanisms. Under conditions of soil salinity, Na uptake by roots is a passive process mediated by non‐selective cation channels (NSCC; Demidchik and Tester 2002); K acquisition is an active process mediated by high‐affinity K^+^ transporter (HAK) and K^+^ uptake permease (Rubio et al. 2020; Wu et al. 2018). Additionally, climate warming and drying, hydrological fluctuations, nutrient enrichment, biological invasion, and anthropogenic disturbance collectively shape the growth and ion distribution of *S. salsa* in the Yellow River Delta wetlands (Guan et al. 2011; Zhang et al. 2020; Wang et al. 2022; Zhang, Xia, et al. 2023; Zhang, Zhang, et al. 2023; Zhang, Shi, et al. 2024). Soil salinity is known to critically influence plant performance and ion partitioning of *S. salsa* (Zhao et al. 2022; Li, Zhang, et al. 2024; Luo et al. 2025), and high Na content can also promote soil alkalinization, structural degradation, and reduced permeability, ultimately impairing plant growth and ionic balance in *S. salsa* (Jiménez‐Ballesta et al. 2024; Kaleem et al. 2024; Sidhoum et al. 2025). Hence, it can be hardly expected that the Na‐K stoichiometry in plants will match that in soil, given these differences in underlying mechanisms.

The decoupling of soil–plant Na relationships may further arise from active ion redistribution within the plant. High leaf Na content in *S. salsa* amplifies tissue‐level heterogeneity, leading to mismatched soil‐tissue correlations (Adams and Shin 2014; Lugo et al. 2024), an effect often overlooked under the homogenization assumption applied in earlier studies (Zhao et al. 2022). Previous studies often assumed a relatively uniform distribution of Na across above‐ground tissues or the whole plant, and consequently reported strong correlations between soil and whole‐plant Na content. These contrasting findings underscore a dialectic between whole‐plant and tissue‐specific perspectives on ion homeostasis. Notably, the strong stem‐soil Na correlation (Figure 2c) likely reflects the stem's role as a transit organ for Na, facilitating ion transfer between roots and leaves (Adams and Shin 2014).

Robust K homeostasis was observed between *S. salsa* and soil in the Yellow River Delta wetlands across most plant tissues, though not in the leaf‐soil relationship (Figures 3d and 9), while no such relationship existed for the Na:K ratio (Figures 4 and 9). Under conditions of sufficient soil K supply, selective K uptake by *S. salsa* coupled with high Na accumulation results in relatively low plant K content (Zhao et al. 2022; Sardans and Peñuelas 2021). Nevertheless, given the critical role of K in plant growth, *S. salsa* has evolved efficient strategies for K utilization and internal distribution (Mori et al. 2011), enabling K homeostasis at both tissues and whole‐plant levels, a key trait supporting adaptation to fluctuating environments (Almeida et al. 2017). Furthermore, we speculate that high leaf Na content may occupy anion‐binding sites in the cytoplasm and vacuoles, partially substituting for K in osmotic functions, thereby limiting K accumulation and contributing to the disrupted soil‐leaf K homeostasis (Almeida et al. 2017; Srivastava et al. 2020). Although the Na:K ratio was negatively correlated with K content, this relationship was primarily driven by variation in Na rather than K. Thus, the decoupling of soil–plant Na dynamics directly governs Na:K ratio homeostasis in *S. salsa* (Zhang, Xia, et al. 2023; Zhang, Zhang, et al. 2023).

Na and K homeostasis, as well as Na:K ratios, were markedly significant within *S. salsa* tissues than between the plant and soil compartments (Figures 2, 3, 4 and 9), and robust Na‐K homeostasis was observed within the multiple plant tissues of *S. salsa* , though not in bulk soil itself (Figures 5 and 9), supporting our third hypothesis that plant internal ion homeostasis is more robust than soil–plant ion coupling. *S. salsa* exhibits autonomous control over Na and K absorption, transport, and partitioning, being a regulatory capacity that underscores its distinctive adaptive strategy compared to abiotic environments (Adams and Shin 2014; Guo et al. 2023). This tightly coordinated Na‐K homeostasis is critical for maintaining essential physiological functions and overall plant viability under variable conditions (Wang et al. 2020; Wegner et al. 2025). Furthermore, *S. salsa* modulates ion dynamics predominantly through internal regulation rather than in response to soil Na‐K stoichiometry, thereby sustaining a dynamic Na‐K balance and reinforcing robust stoichiometric relationships across tissues (Figure 5).

### 4.3 Stem Corresponding Na Stoichiometry and Ion‐Mediated Trade‐Offs Drive Growth Adaptation of *Suaeda salsa* Under High Salinity

**FIGURE 10:**
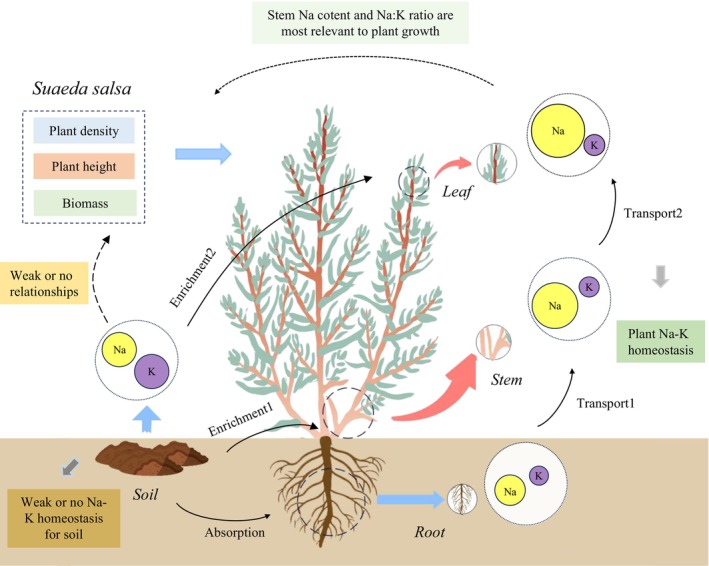
Na‐K stoichiometry and homeostasis across the *Suaeda salsa*‐soil continuum in the Yellow River Delta wetlands.

Plant height was correlated with tissue Na and K contents, and the stem Na content and Na:K ratio emerged as the most significant stoichiometric predictor of growth performance of *S. salsa* (*p* < 0.001, Figures 6, 7, 8, 9, 10), providing partial support for our third hypothesis. Elevated Na levels commonly indicated high soil salinity, under which *S. salsa* enhances population density rather than individual growth, forming a key survival strategy in coastal saline wetlands (Guan et al. 2011; Zhang, Li, et al. 2023). Although increasing soil Na promoted stem Na accumulation in *S. salsa* (Figure 2c), it concurrently reduced plant height and biomass, reflecting a trade‐off strategy at the individual level (Zhao et al. 2022). Notably, K content increased with Na content (Figure 5), suggesting a synergistic ion response that supports osmotic adjustment despite growth reduction. Plant height responded similarly to both tissue K and Na contents, with increasing concentrations of either ion correlating with reduced height—a pattern consistent with the trade‐off between ion accumulation and vertical growth under salinity. This aligns with previous studies indicating that while environmental fluctuations can alter absolute ion concentrations, plants often maintain relatively constant stoichiometric ratios to preserve physiological function (Liu, Ma, et al. 2024; Zhao et al. 2024). Both Na content and Na:K ratio varied considerably across tissues of *S. salsa* and soil layers, yet they were most closely linked to plant density, height, and biomass (Figures 6, 7, 8). This may be attributed to the stem's role as a central hub for structural support, ion storage, and transport (Mishra et al. 2024). Unpublished data indicates that stem account for the largest proportion of biomass in *S. salsa* (Figure S1), affording this tissue greater influence over ion regulation and allocation strategies of Na and K under salinity stress.

This study systematically investigated the Na‐K stoichiometry and their relationships within the soil‐ *S. salsa* system of the coastal wetlands in the Yellow River Delta, and further elucidated how Na‐K stoichiometry influences the growth of *S. salsa* . A key novelty of this work stems from its systemic perspective, which enabled us to delineate the allocation patterns of Na and K across the soil–plant continuum and, from the viewpoint of ion regulation and environmental adaptation, to interpret the strategies employed by *S. salsa* to cope with high‐salinity conditions in coastal wetland habitats. However, the growth of *S. salsa* was only weakly and partially explained by stem Na content and Na:K ratio, as indicated by the relatively low R^2^ values in our models (Figures 6, 7, 8, 9). This limited explanatory power likely arises from the complexity inherent in field conditions, where multiple environmental variables, including but not limited to soil salinity, hydrological fluctuations, climate variability, biological invasions, and anthropogenic disturbances, interact to decisively influence plant growth (Guan et al. 2011; Zhang et al. 2020; Wang et al. 2022; Zhang, Xia, et al. 2023; Zhang, Zhang, et al. 2023; Zhang, Shi, et al. 2024). Moreover, although Na and K are critical ions in salinity stress and ionic homeostasis, they constitute only a subset of the ions and nutrients that govern overall plant performance (Almeida et al. 2017; Zhao et al. 2022). In light of these considerations, several limitations of the present study should be acknowledged. First, our focus was confined to Na and K, without integrating other essential nutrients such as nitrogen and phosphorus; thus, potential multi‐element coupling effects remain unexamined. Second, we did not fully account for the impacts of other environmental drivers, such as hydrological dynamics and ion interactions, on Na‐K stoichiometry in the soil–plant system and subsequent plant growth. Finally, the molecular mechanisms underlying Na and K regulation in *S. salsa* were not addressed, a gap that calls for future cross‐scale investigations. Furtherly, the modest explanatory power of our stoichiometric models highlights the need for future studies to identify and integrate the dominant drivers of growth of *S. salsa* from the multitude of interacting processes operating in costal wetlands of the Yellow River Delta.

## 5 Conclusion

Our study demonstrates that both soil properties and plant tissues of *S. salsa* significantly influence Na‐K stoichiometry. Robust Na, K, Na:K ratio, and Na‐K homeostasis were observed within plant tissues, with more generally weaker homeostasis detected at the soil–plant interface. Specifically, K and Na‐K relationships exhibited stronger regulation between plant and soil compartments compared to Na and Na:K ratios. These findings indicate that *S. salsa* maintains tight internal Na‐K homeostasis across varying soil ion conditions, demonstrating a higher degree of physiological control than soil–plant ion coupling would suggest. Furthermore, stem Na content and Na:K ratio were identified as the strongest predictors of plant growth, while tissue Na and K contents were closely associated with plant height. In contrast, soil Na‐K stoichiometry showed limited utility in predicting plant growth performance. In the future, by conducting systematic research on multiple elements, processes, and scales, we aim to uncover the adaptive mechanisms of *S. salsa* in coastal wetlands and enhance our understanding and predictive ability of wetland ecosystem functions.

## Author Contributions

**Zhang Dongjie:** conceptualization (equal), data curation (equal), writing – original draft (equal), writing – review and editing (equal). **Zhang Kaipeng:** software (equal), writing – original draft (equal), writing – review and editing (equal). **Yang Jingyu:** software (equal), writing – original draft (equal). **Liu Xuepeng:** writing – review and editing (equal). **Zhang Lichao:** investigation (equal), writing – original draft (equal). **He Wenjun:** conceptualization (equal), writing – original draft (equal). **Zhang Mingye:** writing – original draft (equal), writing – review and editing (equal).

## Funding

This work was supported by Shandong Provincial Natural Science Foundation (ZR2024MD072), the Youth Innovation Support Program of Shandong Universities (2023KJ273), the PhD research startup foundation of Binzhou University (No. 2021Y14), and College Student Innovation Training Program Plan (S202410449034).

## Conflicts of Interest

The authors declare no conflicts of interest.

## Supporting information


**Figure S1:** Biomass allocation across root, stem, and leaf tissues of *Suaeda salsa* in the Yellow River Delta wetlands. Different lowercase letters denote significant differences (*p* < 0.05) in biomass among tissues based on one‐way ANOVA with post hoc testing.

## Data Availability

The data presented in this study are available upon request from the corresponding author. The data that support the findings of this study are available in the Supporting Information of this article.
