# Supplementary material for: Stoichiometry and Homeostasis of Sodium and Potassium Underpins Growth Adaptation of Suaeda salsa in the Yellow River Delta Wetland
Source: Ecol Evol. 2026 May 27;16(6):e73687. doi: 10.1002/ece3.73687 (PMC13239276; doi:10.1002/ece3.73687)
Supplement: Supplementary file 1 — Figure S1: Biomass allocation across root, stem, and leaf tissues of Suaeda salsa in the Yellow River Delta wetlands. Different lowercase letters denote significant differences (p < 0.05) in biomass among tissues based on one‐way ANOVA with post hoc testing. [file ECE3-16-e73687-s001.docx]

Supplementary materials





Fig.S1 Biomass allocation across root, stem, and leaf tissues of *Suaeda sals*a in the Yellow River Delta wetlands. Different lowercase letters denote significant differences (*p* < 0.05) in biomass among tissues based on one-way ANOVA with post-hoc testing.
